# PGS/Gelatin Nanocomposite Electrospun Wound Dressing

**DOI:** 10.3390/jcs7060237

**Published:** 2023-06-06

**Authors:** Mahyar Naseri, Aysan Hedayatnazari, Lobat Tayebi

**Affiliations:** 1 School of Dentistry, Marquette University, Milwaukee, WI 53233, USA; 2 Department of Biomedical Engineering, Medical College of Wisconsin, Marquette University, Milwaukee, WI 53233, USA

**Keywords:** electrospun wound dressing, skin tissue engineering, regenerative medicine, gelatin, poly(glycerol sebacate), diabetic wound

## Abstract

Infectious diabetic wounds can result in severe injuries or even death. Biocompatible wound dressings offer one of the best ways to treat these wounds, but creating a dressing with a suitable hydrophilicity and biodegradation rate can be challenging. To address this issue, we used the electrospinning method to create a wound dressing composed of poly(glycerol sebacate) (PGS) and gelatin (Gel). We dissolved the PGS and Gel in acetic acid (75 *v/v*%) and added EDC/NHS solution as a crosslinking agent. Our measurements revealed that the scaffolds’ fiber diameter ranged from 180.2 to 370.6 nm, and all the scaffolds had porosity percentages above 70%, making them suitable for wound healing applications. Additionally, we observed a significant decrease (*p* < 0.05) in the contact angle from 110.8° ± 4.3° for PGS to 54.9° ± 2.1° for PGS/Gel scaffolds, indicating an improvement in hydrophilicity of the blend scaffold. Furthermore, our cell viability evaluations demonstrated a significant increase (*p* < 0.05) in cultured cell growth and proliferation on the scaffolds during the culture time. Our findings suggest that the PGS/Gel scaffold has potential for wound healing applications.

## 1. Introduction

Skin is the body’s largest organ, with multiple critical functions. It serves as a protective barrier against harmful microorganisms and mechanical damage, helps maintain the body’s temperature balance, and aids in the excretion of waste products [1-5]. The integrity of the skin is affected by burns, trauma, and diabetes, which can lead to skin inflammation and bacterial infection.

Wounds, caused by a lack of skin integrity, are a major issue in the world, and have caused economic and social problems in all societies [6-8]. The human skin has the ability to regenerate itself against small and superficial wounds. However, in the case of severe and deep wounds, the natural healing process is not enough, and protection of the injured area is needed until the wound is completely healed. Therefore, the fabrication of proper wound dressing to accelerate the wound healing process is necessary [9]. The ideal wound dressing should perform tasks such as providing a moist environment around the wound, exchanging gas and nutrients, collecting exudate from the wound site, not adhering to the wound, and to prevent bacterial infection and allergic reactions. Accordingly, a wound dressing should be biocompatible, biodegradable, swellable, elastic, and antibacterial. In addition, having a porous structure is necessary for a dressing to improve cell growth, proliferation, migration, and angiogenesis. As a result, the fabrication of an ideal wound dressing to have all these items requires great effort and precision [10,11].

There are various methods, such as solvent casting [12], salt leaching [13], freezedrying [14], and electrospinning [15], in the fabrication of wound dressings. Electrospinning is an effective technique that can use a wide variety of biomaterials to produce nanofibrous structures with uniform and continuous nanofibers and a high specific surface area. This method fabricates highly porous structures, which simplify gas and nutrient exchange and provide moisture around the wound, preventing the excessive drying of the wound. Additionally, electrospun nanofibers have a similar flexibility and tensile strength to human skin and can simulate the extracellular matrix (ECM) structure of the skin tissue, providing a microenvironment which promotes cell attachment, growth, and proliferation [16-18]. Hadizadeh et al. [19] fabricated a poly(ε-caprolactone) (PCL)/gelatin (Gel)-based electrospun scaffold, incorporated with surfactin (Sur) and curcumin (Cur). The PCL/0.2Sur-Gel/3%Cur scaffold showed suitable wettability, mechanical properties, degradation rate, antibacterial activity, and biological properties (in vitro and in vivo). Additionally, Bao et al. [20] fabricated an electrospun scaffold composed of hyaluronic acid (HA), graphene (Gr), and polyphenolic tannic acid (TA). The in vivo results indicated that after 14 days, the wound area of the scaffold loaded with 0.3 *w/v*% TA was 1.12 ± 0.54 mm^2^, which was significantly (*p* < 0.05) better than that of the HA and control groups. However, in addition to the fabrication method of the scaffolds, the biomaterials used for fabricating the scaffolds also have a significant effect on their final properties.

Poly(glycerol sebacate) (PGS) is a tough polyester which is synthesized by the polycondensation of glycerol and sebacic acid. It is a biocompatible, elastic, and biodegradable polyester, and its degradation products can be removed by the body’s metabolism. However, this polymer does not have an ideal hydrophilicity and degradation rate and cannot form a nanofibrous structure due to its low spinnability [21-23]. Thus, it should be blended with hydrophilic polymers.

Gelatin (Gel) is a naturally occurring hydrophilic polymer that can be extracted from collagen, the most abundant protein of the extracellular matrix (ECM). Gel offers several advantages, including biocompatibility, non-immunogenicity, and biodegradability, making it a suitable material for supporting cell attachment, growth, and proliferation. Moreover, Gel is readily available at a relatively low cost. It can be electrospun using aqueous solutions of acetic acid, formic acid, or ethanol [24,25]. In a study, Farahani et al. [26] fabricated a cellulose acetate/gelatin/*Zataria multiflora* nanoemulsion (CA/Gel/ZM-nano) wound dressing using the electrospinning method. The results showed that adding Gel decreased the rate of drug release and increased the cell viability of the dressing. Additionally, in another study, Sanhueza et al. [25] prepared an electrospun scaffold composed of Gel/poly-3-hydroxybutyrate (PHB) nano/microfibers for the healing of diabetic wounds. In vivo results showed that for 14 days, the wound area decreased significantly (*p* < 0.1) after adding Gel to the scaffolds. Moreover, the histological analysis indicated that hypodermis was formed after the treatment of the wounds with the Gel-PHB scaffold.

In this paper, we fabricated and evaluated a PGS/Gel nanofibrous structure using electrospinning to assess its morphological, physical, and biological properties. To the best of our knowledge, this study is the first to examine the fabrication and characterization of a PGS/Gel electrospun scaffold for wound healing applications. Our findings can provide valuable insights for biomedical engineers seeking to develop superior wound dressings with optimal properties.

## 2. Materials and Methods

### 2.1. Materials

The materials used in this study were sourced from various suppliers. Gelatin (Gel) (CAS: 9000-70-8), sebacic acid (CAS: 111-20-6), glycerol (CAS: 56-81-5), dimethyl sulfoxide (DMSO), and 3-(4,5-dimethylthiazol-2-yl)-2,5-diphenyltetrazolium bromide (MTT) were purchased from Sigma Aldrich/USA. N-Hydroxysuccinimide (NHS), 1-Ethyl-3-(3-dimethylaminopropyl) carbodiimide (EDC), acetic acid, and glutaraldehyde (25% aqueous solution) were obtained from Merck/Germany. Phosphate-buffered saline (PBS), Dulbecco’s modified Eagle’s medium (DMEM), GlutaMAX (high glucose), and penicillin/streptomycin were provided by GIBCO/USA. Trypsin-EDTA (0.05% trypsin in 0.04 mM EDTA) was purchased from Bioidea. The human dermal fibroblast (HDF) cell line was acquired from the Cell Bank of the Pasteur Institute.

### 2.2. Fabrication and Crosslinking of Nanofibrous PGS/Gel Scaffolds

**Figure 1.**
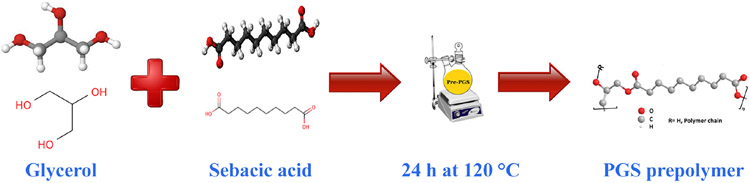
The schematic of the synthesis process of PGS prepolymer.

The PGS prepolymer synthesis method, initially described by Kharaziha et al. [27], was enhanced as follows. To begin, a 1000 mL three-necked flask was utilized, and it was charged with 113.8 g of glycerol and 202.25 g of sebacic acid powder in a 1:1 molar ratio. The flask was then positioned on a magnetic stirrer equipped with a heating system from KI/Germany and subjected to a slow flow of nitrogen gas while maintaining a high vacuum of less than 50 mTorr using equipment from Memmert/Germany. The reactants were stirred magnetically at 120 °C for 24 h. Once the 24 h duration had passed, the nitrogen line was disconnected, and the system was allowed to cool down to room temperature. As a result, a paste-like material, known as the PGS prepolymer, was formed. The schematic representation of this synthesis process can be observed in Figure 1.

For the subsequent crosslinking steps, the method presented by Yoon et al. [28] was followed to synthesize the PGS/Gel copolymer utilizing a previously published polycondensation approach. In brief, varying weight ratios of PGS and Gel (1:1, 2:1, 3:1) were mixed in acetic acid 75% (*v/v*) at a temperature range of 38–42 °C until a homogeneous solution was achieved. The final concentration of the polymer was adjusted within the range of 10–30 wt.%. In order to facilitate the crosslinking process, a crosslinking agent called EDC/NHS was dissolved in ethanol and added to the solution. The mixture was stirred for 30 min to ensure complete crosslinking. The crosslinking process involved optimizing the EDC concentration to 75 mM and selecting a molar ratio of EDC/NHS at 2.5:1. Subsequently, the samples were washed three times with PBS (phosphate-buffered saline) to eliminate any residual crosslinking agent.

Finally, the prepared samples were loaded into a 1 mL plastic syringe equipped with a 21-gauge needle and subjected to electrospinning using an electrospinning device from GMS300/Iran. The concentration of the solution was optimized based on its spinnability and the quality of the nanofibers electrospun on laboratory slides. The electrospinning process was conducted at 25 °C. Following electrospinning, the optimized scaffold was placed in a vacuum oven from Memmert/Germany at room temperature for 6 h to ensure proper drying.

### 2.3. Morphological Assessment

To evaluate the structural characteristics of the scaffolds, such as their shape, porous nature, and overall quality, we employed scanning electron microscopy (SEM, Seron AIS 2300 C/Seron Technologies Inc., Uiwang-si, Republic of Korea). The scaffolds were initially cut into 1 × 1 cm^2^ squares and affixed onto a grid. To enhance their conductivity, a thin layer of gold was deposited onto the scaffold surface. The SEM images of the scaffolds were then captured.

Using Image J software 1.44p (developed by Wayne Rasband at the National Institute of Health/USA), we analyzed the SEM micrographs to measure the diameter of 100 individual fibers within the scaffolds. This process allowed us to determine the range of fiber diameters present. In addition, MATLAB (R2017b) software was utilized to calculate the porosity of the scaffolds based on the information extracted from the SEM images [29].

### 2.4. Fourier Transform Infrared Spectrometry

To comprehensively investigate potential interactions between the two polymers and the chemical structure of the scaffolds, Fourier transform infrared spectroscopy (FTIR) was employed as a testing method. The JASCO FT/IR-6300 instrument from Japan (JASCO Corporation, Tokyo, Japan) was specifically utilized for this purpose.

The FTIR test was conducted at room temperature, utilizing a wavenumber range spanning from 400 to 4000 cm^−1^. Following the electrospinning of PGS and PGS/Gel, 10 mg of the resulting scaffolds was taken and subjected to FTIR analysis. This analytical technique allowed for a detailed examination of the molecular composition and interactions present within the scaffolds. By analyzing the infrared spectra obtained, we were able to gain valuable insights into the chemical bonds and functional groups present, providing a comprehensive understanding of the structural characteristics of the scaffolds and the interactions between the polymers used in their fabrication.

### 2.5. Measurement of Contact Angle

To evaluate the hydrophilicity of the scaffolds, a contact angle meter (XCA-50, PMC/Tehran, Iran) was employed. The contact angle is a widely used parameter to determine the wettability of a material’s surface. The measurement of the water contact angle (WCA) was performed using this instrument.

The analysis took place at room temperature to ensure consistent environmental conditions throughout the experiment. The sessile drop technique, a commonly employed method for measuring contact angles, was utilized. This technique involves placing a small droplet of a liquid on the surface and observing the angle formed between the liquid–air interface and the solid surface.

For this experiment, a droplet volume of 4 μL (microliters) was carefully dispensed onto the scaffolds. The measurement of the contact angle was conducted after allowing 15 s for the droplet to settle on the surface of the scaffolds.

Distilled water was chosen as the liquid for these measurements due to its consistent properties and cleanliness. By observing the contact angle formed by the water droplet on the scaffold surface, valuable information about the hydrophilicity of the scaffolds could be obtained. A smaller contact angle indicates a higher degree of hydrophilicity, suggesting that the surface has a greater affinity for water molecules.

Overall, this comprehensive approach utilizing the contact angle meter, the sessile drop technique, and distilled water as the testing liquid allowed a thorough assessment of the hydrophilicity of the scaffolds at room temperature.

### 2.6. In Vitro Degradation

An in vitro degradation test was conducted to assess the degradation rate of the scaffolds, following the ASTM F1635 standard guidelines. The test aimed to evaluate the changes in the weight of the samples over a specified period. The procedure involved the following steps:

1. Sample preparation: The scaffolds were cut into squares measuring 1 × 1 cm^2^ to ensure uniformity. The initial weight of each sample (W0) was measured using a laboratory scale with a precision of up to 5 decimal places (PLS510-3A/Balingen, Germany).
2. Immersion in PBS solution: The samples were immersed in 5 mL of phosphate-buffered saline (PBS) solution. PBS is commonly used in degradation tests as it simulates the physiological conditions.
3. Incubation: The samples, along with the PBS solution, were placed in an incubator (Memmert/Schwabach, Germany) set to maintain a temperature of 37 °C. This temperature mimics the body’s internal environment.
4. Sampling time points: On specific days during the incubation period (1, 4, 7, 14, and 21), the samples were removed from the solution.
5. Salt removal and drying: To eliminate any salts that had formed on the surface of the samples during incubation, they were rinsed with distilled water. Afterwards, the samples were dried in a vacuum oven (Memmert/Schwabach, Germany) at 37 °C for 2 h.
6. Weight measurement: The dried samples were weighed (Wt) using the same laboratory scale as before.
7. Weight loss calculation: The weight loss percentage was determined using the following formula:

(1) $$\text{Weight loss}\%=\frac{W_{0}-W_{t}}{W_{0}}$$

This formula calculates the percentage of weight lost by the samples during the degradation process.

To ensure accurate results, the buffer solution was completely replaced every 24 h. This step aimed to prevent the accumulation of degradation products in the solution, which could potentially interfere with the weight loss profile of the samples.

By following this comprehensive methodology, the study aimed to provide insights into the degradation rate of the scaffolds, which is a crucial factor in assessing their suitability for biomedical applications.

### 2.7. In Vitro Cellular Studies

After cutting the scaffolds into circles with a diameter of 1.5 cm, they underwent sterilization using a series of treatments. First, the scaffolds were exposed to UV light to eliminate any potential contaminants. Following UV sterilization, they were treated with ethanol, a commonly used disinfectant. Subsequently, the scaffolds were rinsed with phosphate-buffered saline (PBS) to remove any residual ethanol.

Once sterilized, the scaffolds were carefully placed in the wells of 24-well plates, in preparation for cell seeding. Human dermal fibroblast (HDF) cells were prepared by transferring them to culture flasks containing Dulbecco’s Modified Eagle Medium (DMEM) supplemented with 10% fetal bovine serum (FBS) and 1% penicillin–streptomycin. The flasks were then placed in a carbon dioxide (CO_2_) incubator set at 37 °C, with a CO_2_ concentration of 5%. The cells were allowed to grow and proliferate for a period of 3 weeks, with the culture medium being replaced every 2 days until the cell confluency reached 80%.

Upon reaching 80% confluency, the cells were trypsinized and counted. Subsequently, 5 × 103 cells were seeded onto each prepared scaffold, and the plates were returned to the incubator for further incubation. The culture medium was renewed every other day throughout the entire cell culture period.

To assess the viability of the cells cultured on the scaffolds, the MTT assay was performed following the guidelines of the ISO-10993-5 standard. On the 3rd and 5th days of cell culture, the culture medium was removed, and the cells were washed twice with PBS to remove any non-viable cells. Following the washing step, each well received 200 μL of serum-free culture medium containing 20 μL of MTT solution (5 mg/mL). The plates were then incubated at 37 °C and 5% CO_2_ for 4 h. During this time, living cells containing mitochondrial enzymes converted the MTT solution into insoluble blue crystals called formazan, resulting in a change in the color of the medium from yellow to dark blue.

To dissolve the formazan crystals, 200 μL of dimethyl sulfoxide (DMSO) solution was added to each well, replacing the MTT solution. The pipetting process ensured complete dissolution of the crystals. The resulting formazan solution was transferred to a 96-well plate, and the absorbance was measured at a wavelength of 570 nm using a microplate reader (BioTek-FLx800/Paramus, NJ, USA). This measurement allowed an assessment of cell viability to take place, with the results compared to a control sample that lacked scaffolds.

In order to observe cell attachment and proliferation, the cells were cultured on the scaffolds for up to 5 days. On the 5th day of incubation, the culture medium was removed, and the samples were washed three times with PBS. To fix the cells, a solution of 4% glutaraldehyde was applied to the samples, which were then kept at 4 °C for 2 h. After fixation, the scaffolds underwent dehydration using a series of ethanol solutions with concentrations ranging from 50% to 100% (*v/v*). Subsequently, the scaffolds were dried at room temperature. Finally, the assessment of cell attachment and proliferation was performed using scanning electron microscopy (SEM) analysis, specifically using the Seron AIS 2300 C/Seron Technologies Inc., Uiwang-si, Republic of Korea SEM equipment.

### 2.8. Statistical Analysis

Using SPSS software (*n* = 3; version 22), a statistical analysis was conducted to evaluate the data. The analysis involved performing a one-way analysis of variance (ANOVA). The sample size used for the analysis was *n* = 3.

The results of the analysis were reported using the mean ± standard deviation (SD) format. This format provides information about the central tendency of the data (mean) and their variability (standard deviation). The mean value represents the average value of the data points, while the standard deviation reflects the spread or dispersion of the data around the mean.

Furthermore, the significance level for the analysis was set at *p* < 0.05. This indicates that statistical significance was determined based on whether the *p*-value associated with the analysis was less than 0.05. If the *p*-value was found to be less than 0.05, it was considered statistically significant, suggesting that the observed differences in the data were unlikely to have occurred by chance.

In summary, the data analysis was conducted using SPSS software (*n* = 3; version 22) through a one-way ANOVA. The results were reported as mean ± standard deviation (SD), and the significance level was defined at *p* < 0.05.

## 3. Results

### 3.1. Optimization of Electrospinning Parameters

In the fabrication of polymer-based fibers using the electrospinning method, several parameters significantly affect the surface morphology and diameter of the fibers. These parameters can be broadly categorized into two groups: solution parameters and process parameters. By understanding and controlling these factors, researchers can optimize the electrospinning process to obtain desired fiber characteristics.

Solution parameters refer to properties related to the polymer solution used in electrospinning. Some examples of solution parameters include solution viscosity, concentration, solvent volatility, and the molecular weight of the polymers. These factors play a crucial role in determining the spinnability of the solution and the resulting fiber morphology. For instance, higher solution viscosity and concentration generally lead to the formation of thicker fibers, while lower viscosity and concentration result in thinner fibers. The choice of solvent and its volatility affects the drying rate of the polymer solution during electrospinning, influencing the fiber diameter and surface morphology. Additionally, the molecular weight of the polymers affects their chain entanglement and solution properties, which in turn impact fiber formation.

On the other hand, process parameters are related to the specific electrospinning setup and conditions. They include applied voltage, needle-to-collector distance, and solution flow rate. The applied voltage determines the electrostatic force experienced by the polymer solution, affecting the stretching and elongation of the polymer jet as it is ejected from the spinneret. The needle-to-collector distance determines the travel distance of the polymer jet before reaching the collector surface, which affects the fiber alignment and deposition pattern. The solution flow rate controls the rate at which the polymer solution is dispensed from the spinneret, influencing the fiber diameter and uniformity.

Our experimental results have shown certain trends and limitations regarding these parameters. For instance, electrospinning does not occur when the polymer concentration is below 30 wt.%, resulting in large drops of the solution being sprayed onto the collector. Conversely, at polymer concentrations above 30 wt.%, only short, irregular, and thick fibers are electrospun. Furthermore, specific ratios of polymer blends, such as PGS/Gel at 2:1 and 1:1, do not exhibit spinning behavior. However, a polymer ratio of 3:1 has been found to produce regular and uniform fibers. Consequently, the optimal conditions identified in this research include a polymer concentration of 30 wt.% with a PGS/Gel ratio of 3:1.

In addition to the solution parameters, other optimal electrospinning parameters are determined in our study. These include a needle-to-collector distance of 15 cm, an applied voltage of 20 kV, and a solution flow rate of 0.5 mL/h. These parameters have been identified as suitable for achieving the desired fiber characteristics in the electrospinning process.

### 3.2. Morphological Assessment

**Figure 2.**
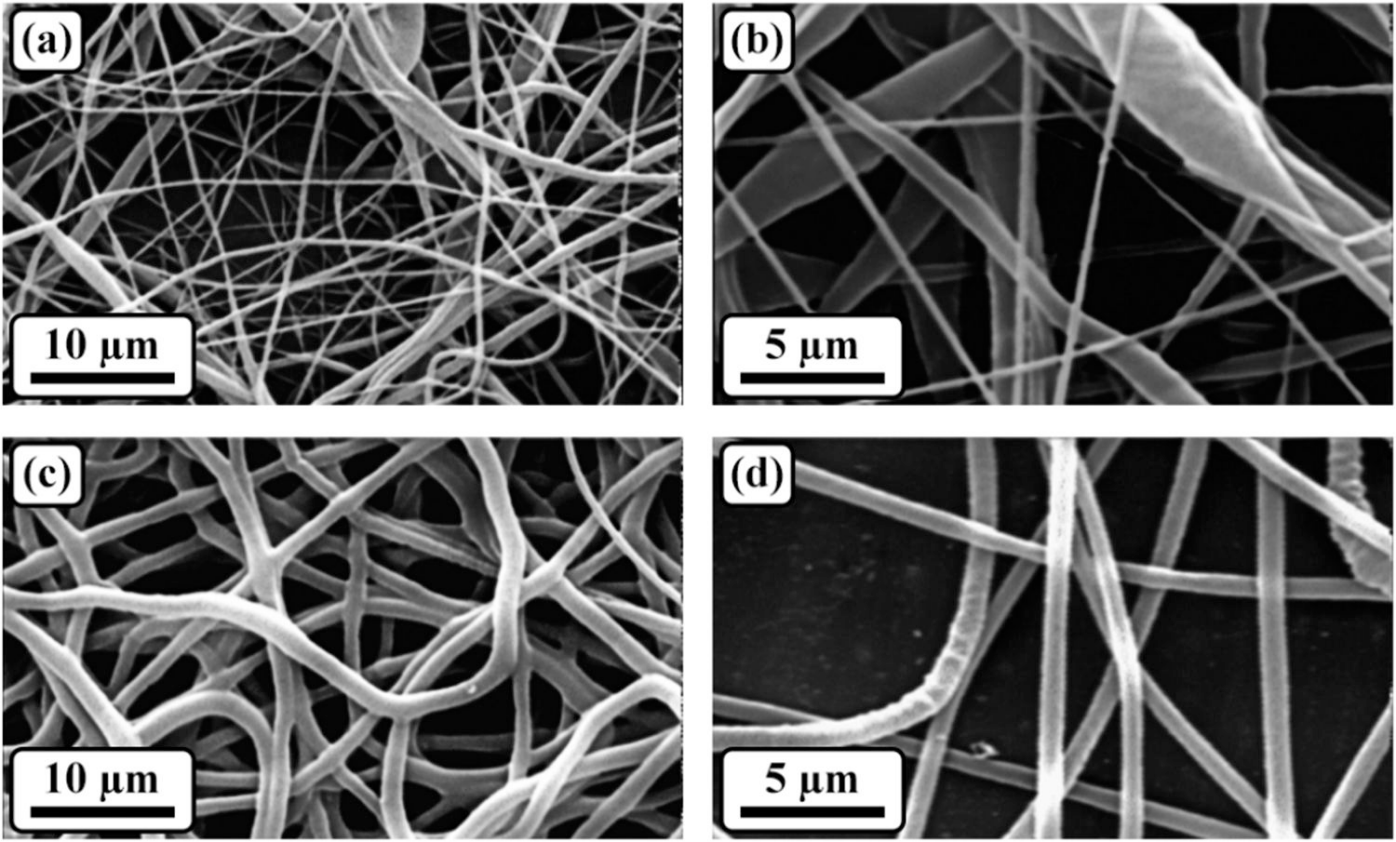
The SEM micrographs of (**a,b**) PGS and (**c,d**) PGS/Gel.

The morphological properties of the scaffolds were thoroughly investigated through SEM analysis, as illustrated in Figure 2. The obtained micrographs of the electrospun samples revealed a distinct structure characterized by bead-less nanofibers with a porous arrangement. Notably, when comparing the samples with and without gelatin, it was observed that the addition of gelatin led to a more uniform distribution of the fiber diameter. Specifically, the sample containing gelatin exhibited an average fiber diameter of 252.4 ± 32.5 nm.

To quantify the porosity of the scaffolds, SEM micrographs were analyzed using MATLAB software. The results revealed that all the samples possessed a porosity level exceeding 75%. This high porosity is a desirable characteristic, particularly in the context of wound dressing applications. Importantly, the interconnected nature of the porosity was maintained both before and after the crosslinking process, further enhancing the scaffold’s suitability for wound healing.

To provide a comprehensive overview of the experimental findings, the average fiber diameter and porosity percentage of the samples have been summarized in Table 1. These quantitative results serve to support and complement the qualitative observations made through SEM analysis.

Overall, the SEM analysis provided valuable insights into the morphological properties of the scaffolds, highlighting the presence of a porous nanofiber structure and the impact of gelatin on fiber diameter uniformity. The interconnected and highly porous nature of the scaffolds makes them promising candidates for wound dressing applications. Further details regarding the specific values of the fiber diameter and porosity percentage can be found in Table 1.

### 3.3. Structural Evaluation

FTIR spectroscopy was utilized to analyze the interactions between PGS and Gel after the crosslinking process. The aim was to investigate the blend interactions and compare the FTIR spectra of PGS and PGS/Gel scaffolds.

**Figure 3.**
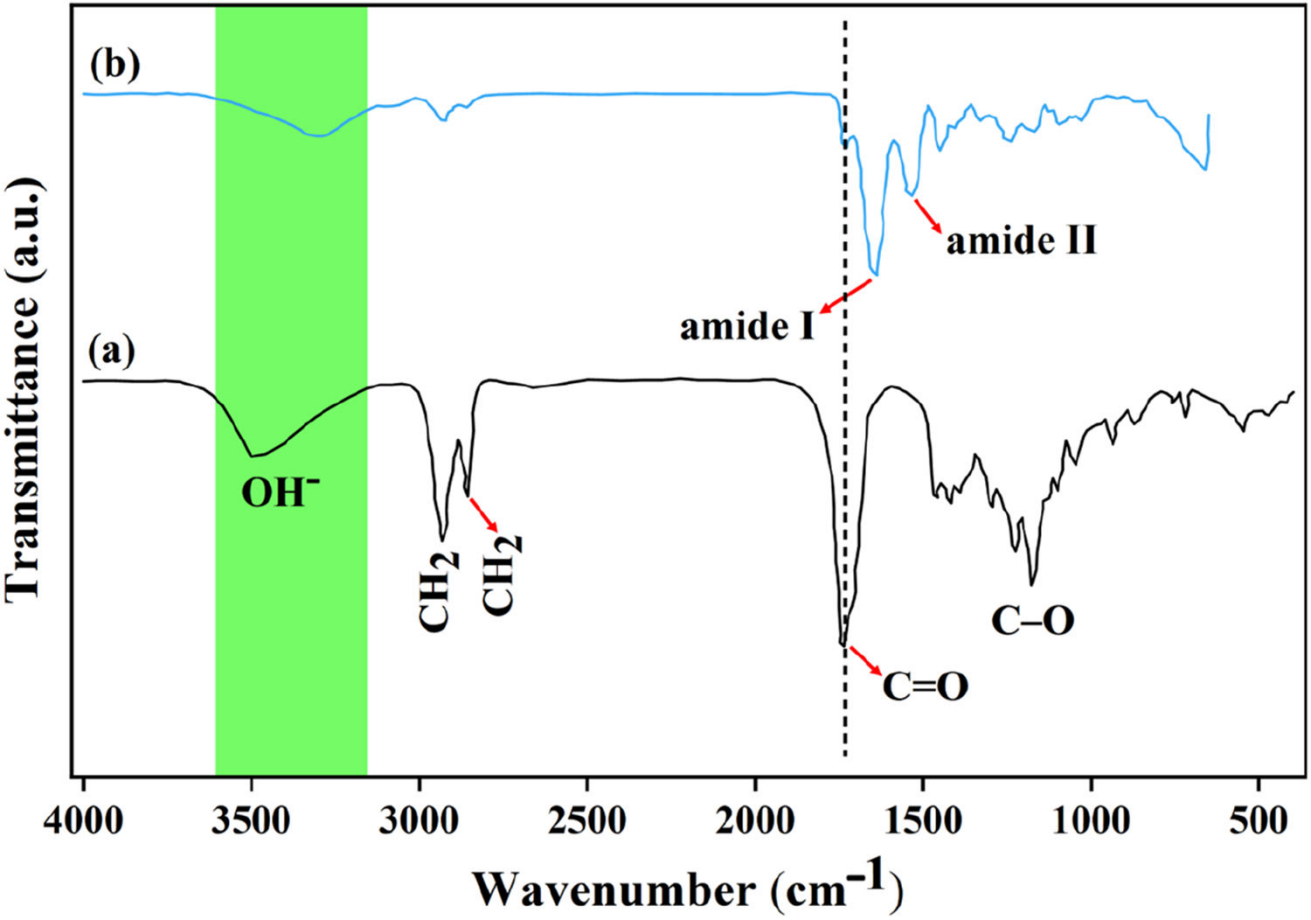
The FTIR spectra of (**a**) PGS and (**b**) PGS/Gel.

In Figure 3a, the FTIR spectrum of PGS exhibited characteristic peaks that provided information about its chemical composition. Two peaks observed at 2855 cm^−1^ and 2933 cm^−1^ were assigned to the CH2 groups present in PGS. Additionally, peaks at 1177 cm^−1^ and 1735 cm^−1^ indicated the formation of C-O and C═O bonds, respectively, confirming the successful synthesis of PGS. The intense peaks observed in the range of 3100–3600 cm^−1^ were attributed to the presence of OH- functional groups in PGS [30,31].

The synthesis of PGS involves a chemical reaction between glycerol, a simple polyol compound, and sebacic acid, which contains carboxyl groups. The sterilization process combines three hydroxyl groups from glycerol with two carboxyl groups from sebacic acid. The characteristics of the resulting polymer vary depending on the ratio of glycerol to sebacic acid used.

Moving on to the FTIR spectrum of PGS/Gel (Figure 3b), the presence of Gel nanofibers was observed. Peaks corresponding to amide bonds were detected at 1533 cm^−1^ and 1637 cm^−1^. In the hybrid mat, these peaks exhibited slight shifts and were observed at 1728 cm^−1^ for C═O bonds and at 1545 cm^−1^ and 1650 cm^−1^ for amide bonds [31,32]. The prominent bands observed in the characteristic peaks corresponded to amide I and amide II, which indicated the presence of hydrogen bonding between the free O-H and N-H groups and the carbonyl group of the peptide linkage within the protein. The convergence of these bands around 3500/cm suggested the potential presence of hydrogen bonding.

The molar ratio of EDC to Gel ε-amino groups influenced the extent and percentage of crosslinking. Increasing the molar ratio led to a higher number of ε-amino groups participating in the crosslinking process. This was supported by the disappearance of sebacic acid bands at 940 cm^−1^ and 1311 cm^−1^, along with the shift of the carbonyl (C═O) band to a lower wavenumber, confirming the involvement of the PGS polymer in the crosslinking process [32].

### 3.4. Hydrophilicity Assessment

**Figure 4.**
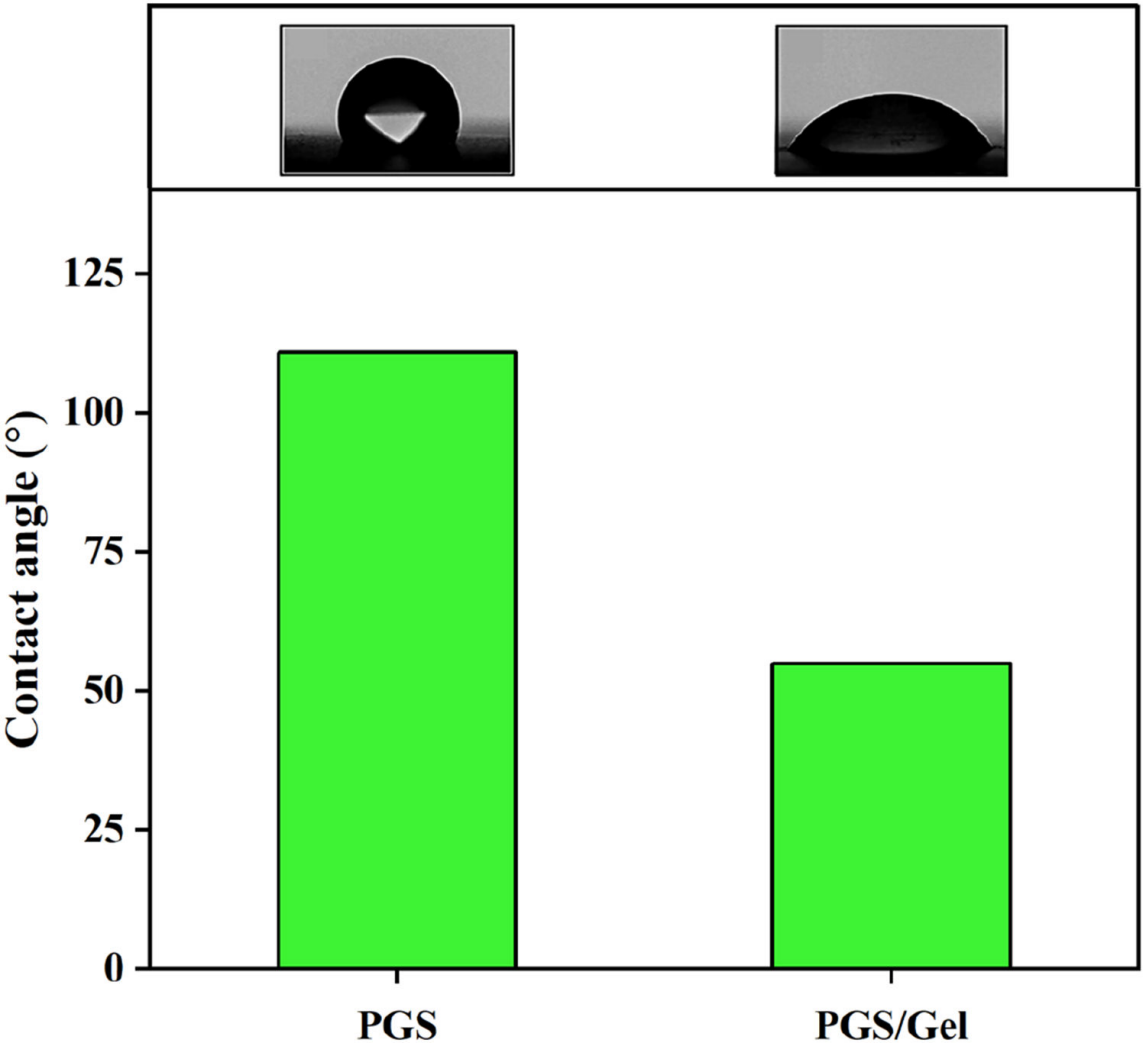
The water contact angle measurements of the samples.

Contact angle measurements were utilized as a means to assess the hydrophilicity of the scaffolds. Hydrophilic surfaces typically exhibit contact angles below 90°, while hydrophobic surfaces tend to display contact angles above 90°. In this study, Figure 4 illustrates that the contact angle of the scaffolds decreased from an initial value of 110.8° ± 4.3° to 54.9° ± 2.1° upon the addition of gelatin, indicating a shift towards hydrophilicity.

Moreover, the findings demonstrated that the PCS/Gel fibrous scaffolds possessed significantly improved wettability compared to PGS alone. This enhancement in wettability can be attributed to the fabrication process, which involved reducing the scaffold diameters and increasing the specific surface area. Consequently, the resulting scaffolds exhibited a more hydrophilic nature, as evidenced by the reduced contact angle of water on their surfaces. This increased hydrophilicity rendered the scaffolds more favorable for cellular applications as it promoted cell contraction and enhanced their suitability for use in various cell-based processes.

### 3.5. In Vitro Degradation

**Figure 5.**
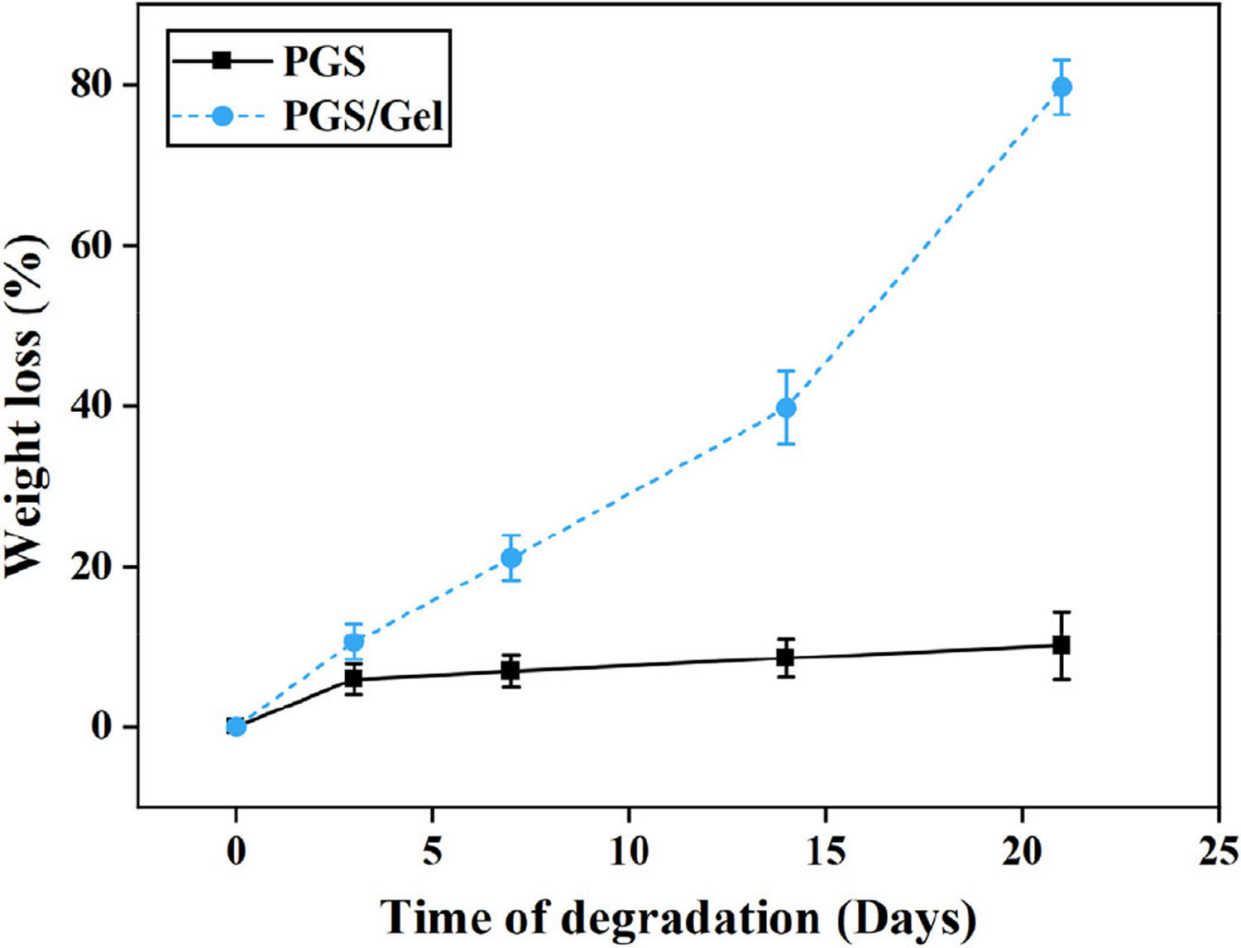
The weight loss measurements of the samples immersed in the PBS solution.

The weight loss measurements were conducted to assess the degradation characteristics of the scaffolds. Figure 5 illustrates the results obtained from the study. Initially, a pure PGS scaffold was tested, and it exhibited a weight loss of only 10.1 ± 4.2% after 21 days.

To enhance the biodegradation process, gelatin was incorporated into the scaffold. The addition of gelatin significantly increased the rate of degradation, resulting in the weight loss of the scaffold reaching 79.7 ± 3.4% of its initial weight during the same 21-day period. This substantial increase in weight loss indicates that the presence of gelatin promotes the faster degradation of the scaffold.

The degradation mechanism of PGS scaffolds primarily revolves around the breakdown of ester bonds, leading to the formation of sebacic acid and glycerol monomers. The number of ester bonds present in the PGS/Gel scaffold is comparatively lower, which consequently accelerates the degradation process of the scaffold.

In summary, the study demonstrates that the incorporation of gelatin into PGS scaffolds enhances their biodegradation behavior. The weight loss measurements indicate that the degradation rate is significantly increased when gelatin is present, which is attributed to the faster decomposition of ester bonds. These findings contribute to a better understanding of the factors influencing the degradation kinetics of scaffolds and provide insights for the design and development of bioresorbable materials in various applications.

### 3.6. In Vitro Cellular Studies

**Figure 6.**
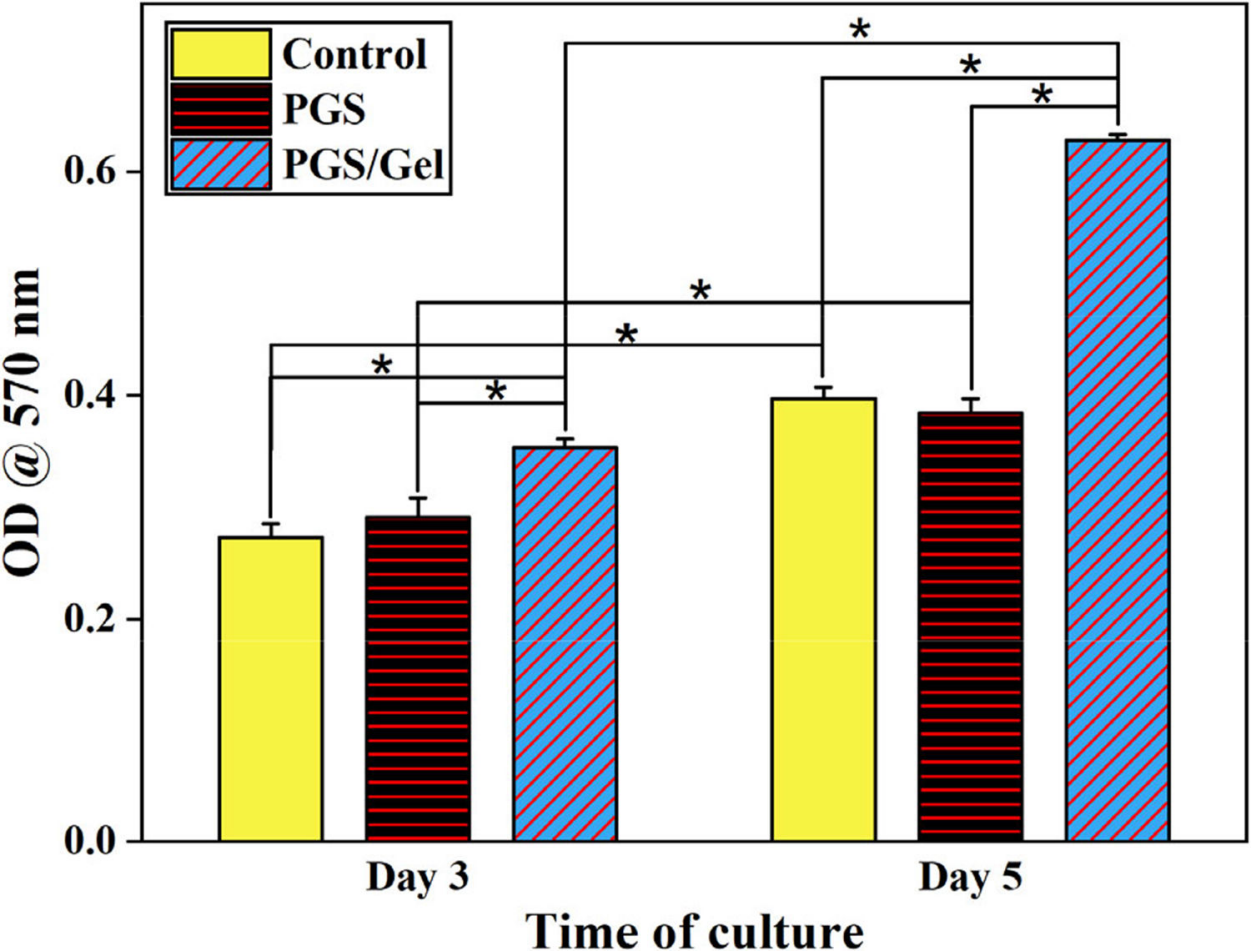
The MTT results of the samples (* indicate *p* value < 0.05).

The cell viability of the scaffolds was assessed using the MTT assay, which provides insights into the metabolic activity and overall health of the cells. The results of this assay are presented in Figure 6, illustrating the survival rate of cells cultured on two types of scaffolds: PGS and PGS/Gel. The cells were incubated for 3 and 5 days, and their viability was evaluated at each time point.

The incorporation of gelatin, a natural polymer, into the scaffold formulation resulted in noticeable improvements in cell proliferation across all the tested samples throughout the entire 5-day period. This indicates that the presence of gelatin positively influenced the growth and division of cells on the scaffolds, suggesting its potential for promoting tissue regeneration.

When comparing the viability of cells cultured on the pure PGS scaffold to the control sample (scaffold-free), no significant difference was observed (*p* > 0.05). This implies that the cell viability on the pure PGS scaffold was comparable to that of cells cultured without any scaffold. Therefore, the pure PGS scaffold did not have a negative impact on cell survival.

On the other hand, the PGS/Gel scaffold demonstrated significantly increased cell viability (*p* < 0.05) compared to the control sample. This finding indicates that the incorporation of gelatin as a hydrophilic segment in the formulation of the PGS scaffold significantly enhanced the hydrophilicity of the scaffolds. As a result, the improved hydrophilicity facilitated enhanced interactions between the cells and the scaffold, leading to improved cell viability and proliferation.

**Figure 7.**
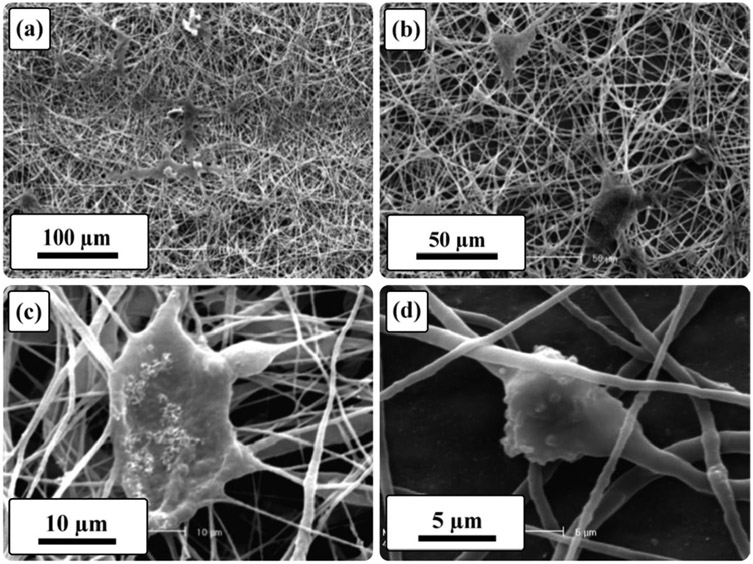
The SEM micrographs of the PGS/Gel scaffold on the 5th day of cell culture at (**a**) 250×, (**b**) 500×, (**c**) 2000×, and (**d**) 4000× magnifications.

To further investigate the cell attachment on the PGS/Gel scaffold, SEM was employed on the 5th day of cell culture, and the micrographs are presented in Figure 7. According to the results, the SEM images confirm that the PGS/Gel scaffold supported the attachment and spreading of fibroblast cells. This observation provides additional evidence that the introduction of gelatin in the formulation of the PGS scaffold promoted favorable cell–substrate interactions, facilitating the adhesion and growth of cells on the scaffold surface.

In summary, the MTT assay results indicate that the incorporation of gelatin as a natural polymer component in the PGS scaffold formulation improved cell proliferation. The pure PGS scaffold did not negatively affect cell viability, while the PGS/Gel scaffold exhibited significantly increased cell viability compared to the control sample. The enhanced hydrophilicity resulting from the inclusion of gelatin likely played a crucial role in improving cell–substrate interactions. Moreover, the SEM images on the 5th day of culture confirmed that the PGS/Gel scaffold supported cell attachment and spreading, further validating its suitability for cell growth in tissue engineering applications.

## 4. Discussion

Fiber uniformity and integrity are crucial morphological factors in the development of electrospun scaffolds. They play a significant role in providing an appropriate surface that promotes cell attachment and proliferation [33,34]. The incorporation of Gel into the scaffold fabrication process has been found to enhance the production of a more uniform nanofibrous structure. This improvement is likely attributed to the formation of hydrogen bonds between PGS and Gel [35]. Similar hydrogen bonding interactions have been observed in other studies, such as the confirmation of hydrogen bond formation between Gel and poly(3-hydroxybutyrate-co-4-hydroxybutyrate) (P3,4HB) polyester [36].

In addition to fiber morphology, the porosity percentage is another crucial factor to consider in scaffold design. A three-dimensional scaffold with a porosity percentage above 70% exhibits an interconnected structure that allows cells to penetrate its depth, facilitating nutrient and waste exchange [37]. Our fabricated scaffolds demonstrate porosity percentages above 75%, indicating a suitable three-dimensional structure for various biomedical applications. The FTIR results reveal a shift of the carbonyl functional group peak towards lower wavenumbers and a decrease in its intensity, suggesting the formation of hydrogen bonds between the carbonyl groups of PGS and the amide groups of Gel. Furthermore, hydrogen bonds have been observed between Gel and polycaprolactone (PCL) in an electrospun multilayer nanofibrous scaffold in another study [38].

Surface properties, particularly hydrophilicity, play a vital role in promoting cell attachment, spreading, and proliferation. A water contact angle measurement is commonly employed to assess the hydrophilicity of scaffold surfaces [33]. Studies have shown that surfaces with water contact angles ranging from 40° to 80° exhibit appropriate hydrophilicity and can support cell adhesion and proliferation [34,37]. In the case of the PGS/Gel n scaffold, the addition of Gel significantly (*p* < 0.05) reduces the water contact angle, likely due to the hydrophilic nature of Gel resulting from the presence of hydroxyl groups. This finding is consistent with other studies [39,40]. Additionally, the formation of hydrogen bonds within the PGS/Gel scaffold (as indicated by FTIR results) contributes to the increased hydrophilicity, as demonstrated in previous research [33].

Considering the biodegradation rate, an ideal scaffold should match the rate of the wound healing process [37]. Electrospun structures, with their high porosity and nano-sized fibers, possess a relatively large surface area, making them suitable for use as biodegradable scaffolds [34]. In vitro biodegradation analysis reveals that pure PGS scaffolds exhibit very slow degradation rates over 21 days due to their hydrophobicity, which is not desirable for wound healing applications [41]. However, the addition of Gel increases the biodegradation rate of the PGS/Gel scaffold, primarily due to the hydrophilic nature of Gel [38,42,43], as well as the formation of hydrogen bonds between Gel and PGS [34]. The MTT assay results further support the beneficial effects of Gel, as its addition significantly (*p* < 0.05) enhances cell viability on the PGS/Gel scaffold. This improvement can be attributed to the hydrophilic nature of Gel, the presence of sufficient hydroxyl groups on the scaffold’s surface [37], and the formation of hydrogen bonding interactions between PGS and Gel [34].

Moreover, studies have shown that the presence of Gel in scaffold formulations, such as PCL/Gel hierarchical scaffolds, increases cell viability and promotes appropriate cell attachment and proliferation [44]. A microscopic examination, specifically SEM, on the fifth day of cell culture confirmed the favorable cellular behavior observed on the surface of the PGS/Gel scaffolds, indicating their suitability for cardiac tissue engineering applications [27,45].

## 5. Conclusions

Here, the PGS/Gel nanofibrous scaffold was electrospun with an optimal solution concentration and PGS-to-Gel ratio. The optimal PGS-to-Gel ratio and polymer concentration are 3:1 and 30 wt.%, respectively. The PGS/Gel scaffold was fabricated with uniform and continuous nanofibers using a voltage of 20 kV, a flow rate of 0.5 mL/h, and a needle-to-collector distance of 15 cm. SEM results show that the PGS/Gel scaffold has a uniform and porous nanofibrous structure with a fiber diameter of 252.4 ± 32.5 nm. The FTIR results indicate the possibility of the formation of hydrogen bonding between PGS and Gel. In addition, WCA measurements demonstrate that the PGS/Gel scaffold has a WCA of 54.9° ± 2.1°, showing the hydrophilicity of the blend scaffold. In vitro degradation results also show that adding Gel has optimized the rate of degradation. Moreover, MTT assay and cell attachment results indicate the positive effects of adding Gel on the biological properties of the blend scaffold. In conclusion, the results of this study could help biomedical engineers to design and fabricate a wound dressing with optimal properties.

## Figures and Tables

**Table 1.** The average fiber diameter and porosity percentage of the samples.

Sample	Mean Fiber Diameter (nm)	Porosity Percentage (%)
PGS	371.2 ± 64.8	77.2 ± 2.4
PGS/Gel	252.4 ± 32.5	80.8 ± 1.1

## Data Availability

There is no additional data available for this study other than what is reported in the manuscript.
